# Supplementary material for: Oxicam-type non-steroidal anti-inflammatory drugs inhibit NPR1-mediated salicylic acid pathway
Source: Nat Commun. 2021 Dec 15;12:7303. doi: 10.1038/s41467-021-27489-w (PMC8674334; doi:10.1038/s41467-021-27489-w)
Supplement: Supplementary file 1 — Supplementary Information [file 41467_2021_27489_MOESM1_ESM.pdf]

Salicylic acid derivatives

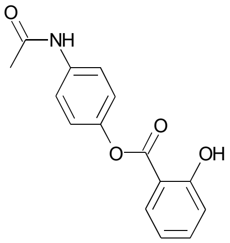

Acetaminosalol

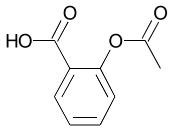

Acetyl salicylic acid (Aspirine)

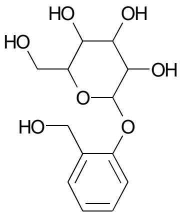

D-(-)-Salicin

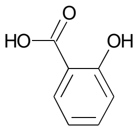

Salicylic acid

Propionic acid derivatives

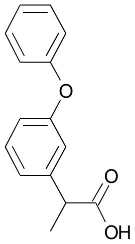

Fenoprofen

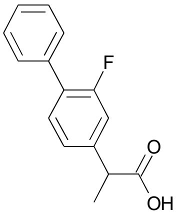

Flurbiprofen

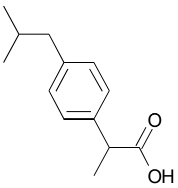

Ibuprofen

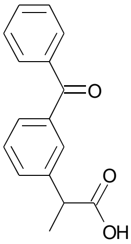

Ketoprofen

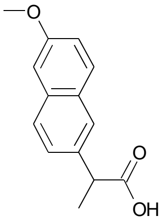

Naproxen

Acetic acid derivatives

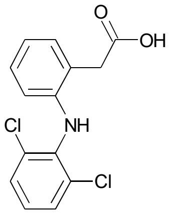

Diclofenac

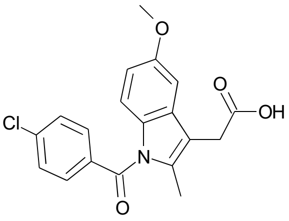

Indomethacin

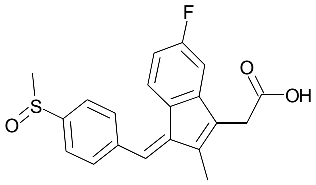

Sulindac

Oxicam derivatives

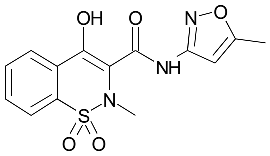

Isoxicam

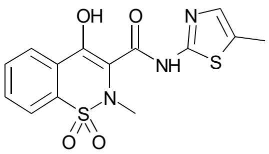

Meloxicam

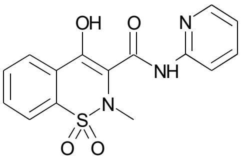

Piroxicam

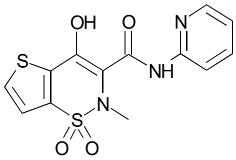

Tenoxicam

Anthranilic acid derivatives

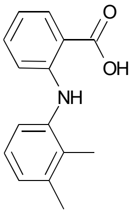

Mefenamic acid

Others

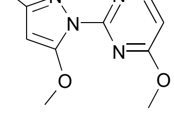

Epirizole

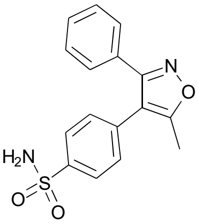

Valdecoxib

Supplementary Fig. 1 Chemical structure of NSAIDs identified by chemical screening.

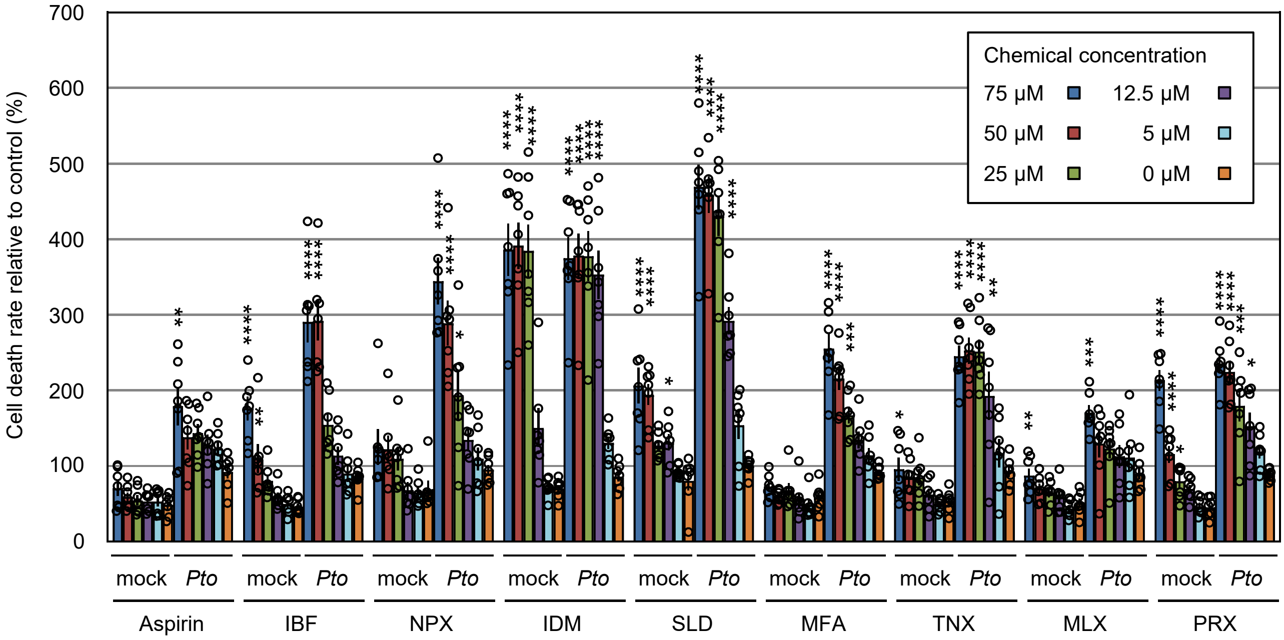

Supplementary Fig. 2 Effect of NSAIDs on *Pto avrRpm1*-induced cell death in *Arabidopsis* suspension cells. *Pto avrRpm1* suspension (final OD_{595} = 0.2) was added to *Arabidopsis* suspension cells after adding compounds (concentrations indicated in the right panel). After 20 hours incubation, dead cells were stained with Evans blue, and cell death was determined. Relative values were calculated based on means from controls (DMSO + *Pto*) for each experimental group. Data are shown as means ± SE (n = 7 biologically independent samples). Asterisks indicate significant differences from DMSO control (0 µM) to each treatment groups (two-sided Dunnett test, *$p < 0.05$, **$p < 0.01$, ***$p < 0.005$, ****$p < 0.0001$).

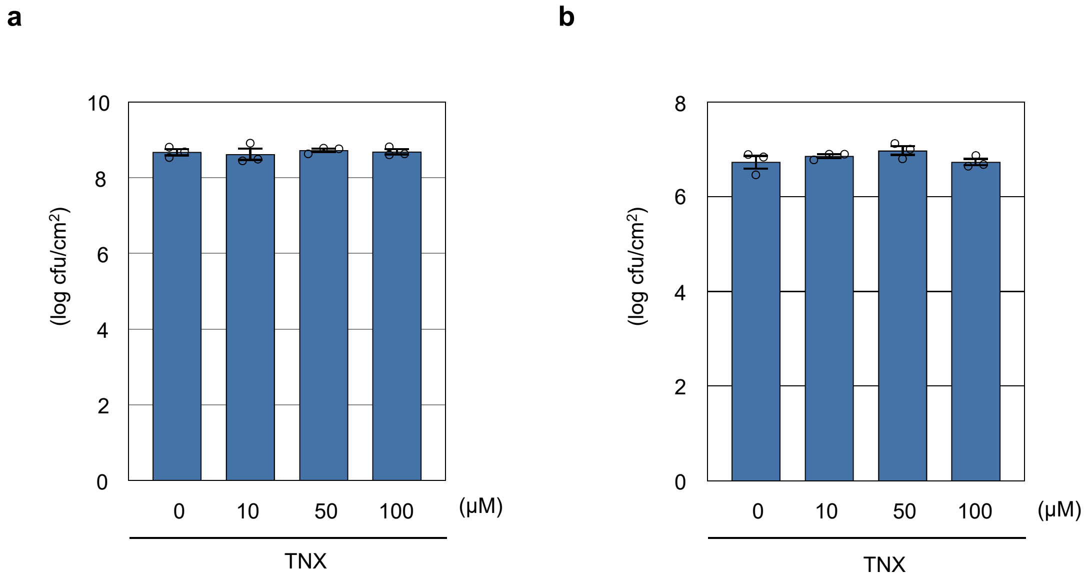

Supplementary Fig. 3 TNX effect on bacterial growth. a, b Bacterial suspension of *Pseudomonas syringae* pv. *tomato* DC3000 (1 x 10^7 cfu/mL) in M9 minimal medium (**a**) or 10 mM $MgCl_2$ (**b**) supplemented with TNX were incubated at 28°C for 38 hours with continuous shaking at 160 rpm. Serial dilution of the cultures were spread onto LB agar media containing kanamycin and rifampicin and the number of colonies were counted. The bars represent the mean ± SE (*n* = 3 biologically independent experiments).

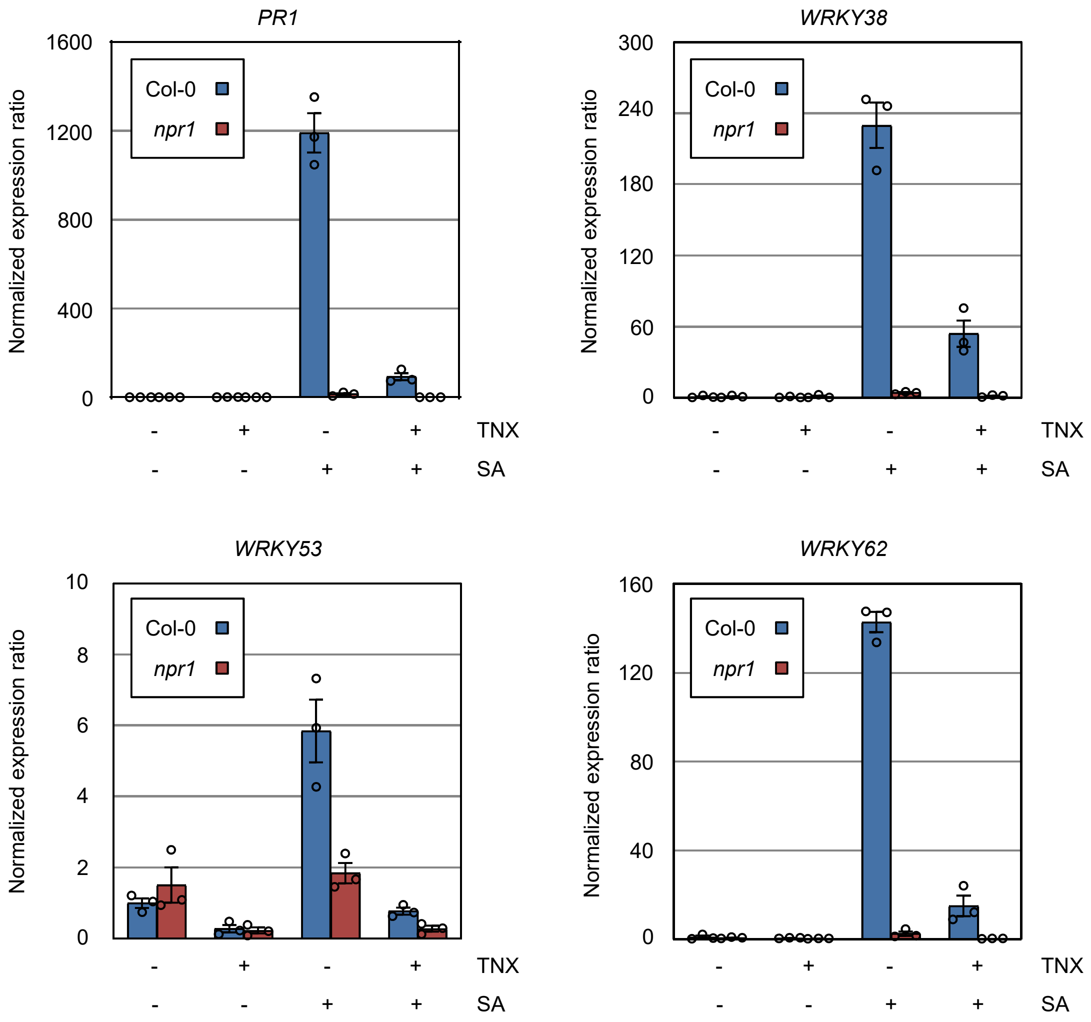

Supplementary Fig. 4 Expression pattern of *PR1*, *WRKY18*, *WRKY39*, and *WRKY62* in the transcriptome analysis. Gene expression values obtained from transcriptome analysis (Supplementary Dataset 1) were normalized with that in mock-treated Col-0 WT. The bars represent the mean ± SE (*n* = 3 biologically independent samples).

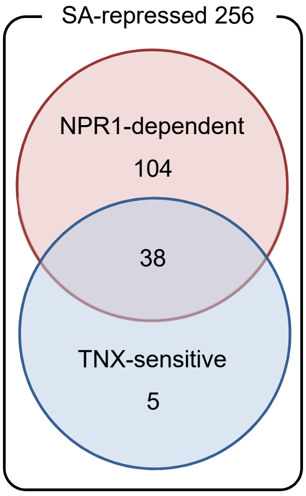

Supplementary Fig. 5 Venn diagram showing the number of SA-repressed genes whose expression levels were reduced in the *npr1* mutant and/or co-treatment with TNX. Comparison between SA- and mock-treated Col-0 WT identified 256 genes which were suppressed by SA (FDR = 0.001 and $\log_2$FC (SA/mock in WT) < -1) and were categorized as "SA-repressed". Among the SA-repressed genes, 142 genes in the *npr1* mutant showed weaker suppression than that observed in Col-0 WT upon SA treatment ($\log_2$FC (SA/mock in WT) – $\log_2$FC (SA/mock in *npr1*) < -1). These genes were classified as "NPR1-depedent". Forty-three genes out of the SA-repressed genes were suppressed by co-treatment with TNX in Col-0 WT (FDR = 0.001 and $\log_2$FC (SA/SA+TNX) < -1) and were classified as "TNX-sensitive".

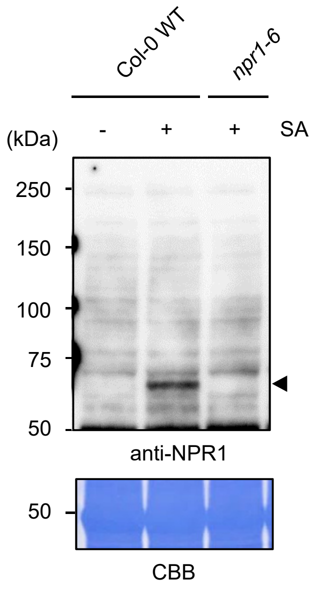

Supplementary Fig. 6 Specific reaction of anti-NPR1 antibody. *Arabidopsis* seedlings of *npr1-6* mutant or Col-0 WT were treated with 100 µM SA, and were sampled 24 hours after treatment. NPR1 protein was detected by immunoblotting using anti-NPR1 antibody. CBB staining were shown as a loading control. Experiments were repeated 3 times with similar results.

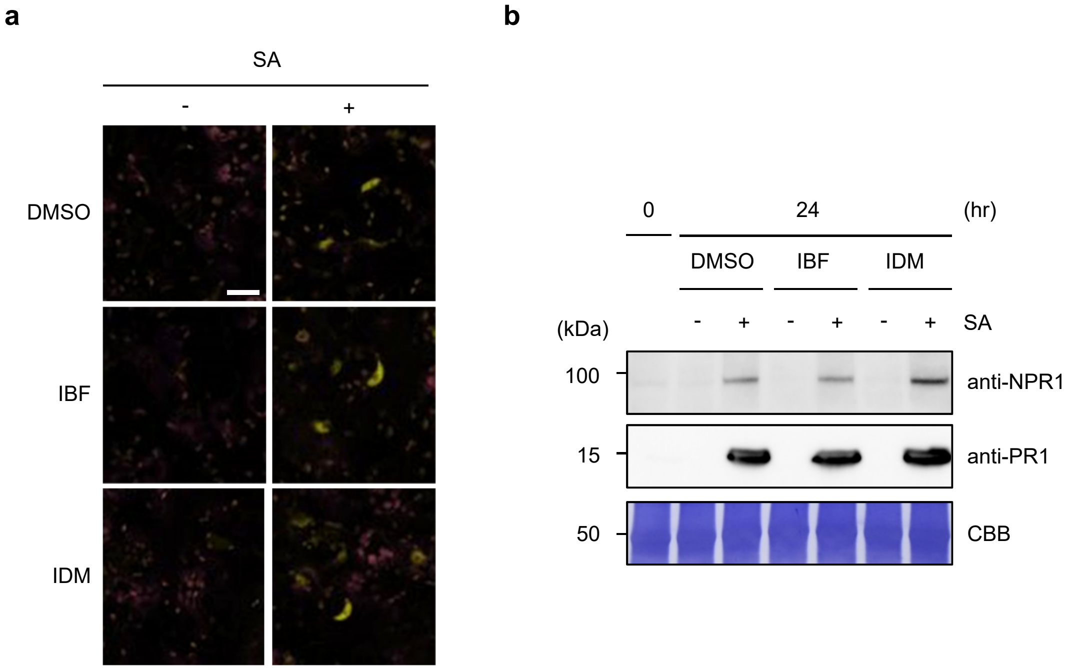

Supplementary Fig. 7 Effect of IBF and IDM on SA-induced NPR1 accumulation. a *NPR1p*:*NPR1-YFP* seedlings were treated with 100 µM NSAIDs (IBF or IDM) or 0.5% DMSO for 1 hour before 100 µM SA (+) or water (-) treatment for 24 hours. Fluorescent image was obtained with a confocal laser microscope. YFP fluorescence and chlorophyll autofluorescence were shown in yellow and magenta, respectively. Bar = 20 µm. **b** *NPR1p*:*NPR1-YFP* seedlings were treated with SA and/or NSAIDs as in (**a**). Protein extracts of the seedlings were resolved by SDS-PAGE and analyzed by immunoblotting using anti-NPR1 and anti-PR1 antibodies. CBB staining was shown as a loading control. Experiments were repeated 3 times with similar results.

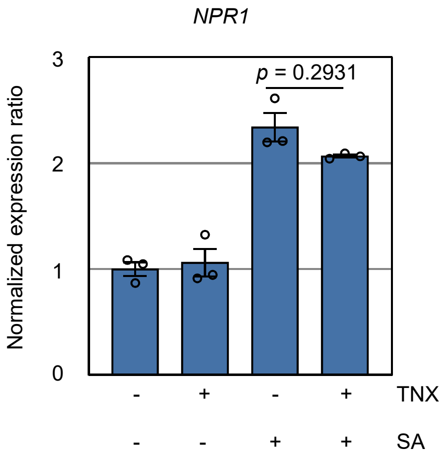

Supplementary Fig. 8 Expression pattern of *NPR1* in the transcriptome analysis. Expression value of *NPR1* obtained from transcriptome analysis (Supplementary Dataset 1) were normalized with that in mock-treated Col-0 WT. The bars represent the mean ± SE (n = 3 biologically independent samples). p-value is from two-sided Tukey-Kramer test.

a

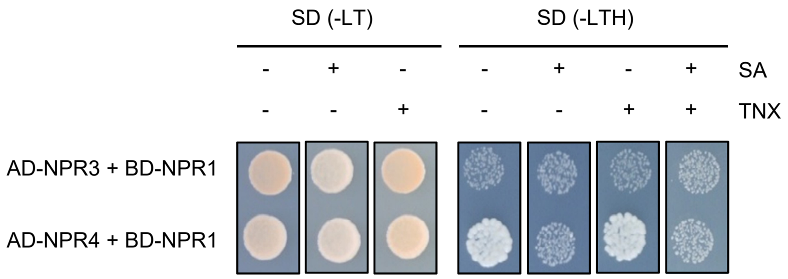

b

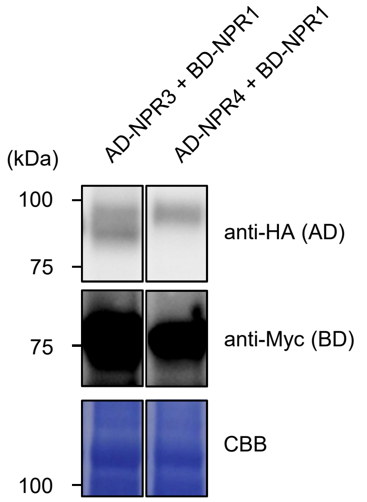

Supplementary Fig. 9 Yeast two-hybrid assay for NPR1-NPR3/4 interaction in the presence of SA and/or TNX. a Yeasts expressing *NPR* genes fused with GAL4 activation domain (AD) or DNA-binding domain (BD) were spotted on synthetic dropout (SD) media lacking Leu and Trp (-LT) or Leu, Trp and His (-LTH) without or with 100 μM SA and/or 100 μM TNX. **b** Immunodetection of GAL4 AD or BD fused NPR proteins. Yeast strains were grown on SD (-LT) plates, and then fresh single colonies were grown in SD (-LT) liquid media for 1 day. Total proteins were extracted from the yeasts and subjected to immunoblot analysis using anti-HA and anti-Myc antibodies. CBB staining were shown as a loading control. Experiments were repeated 3 times with similar results.

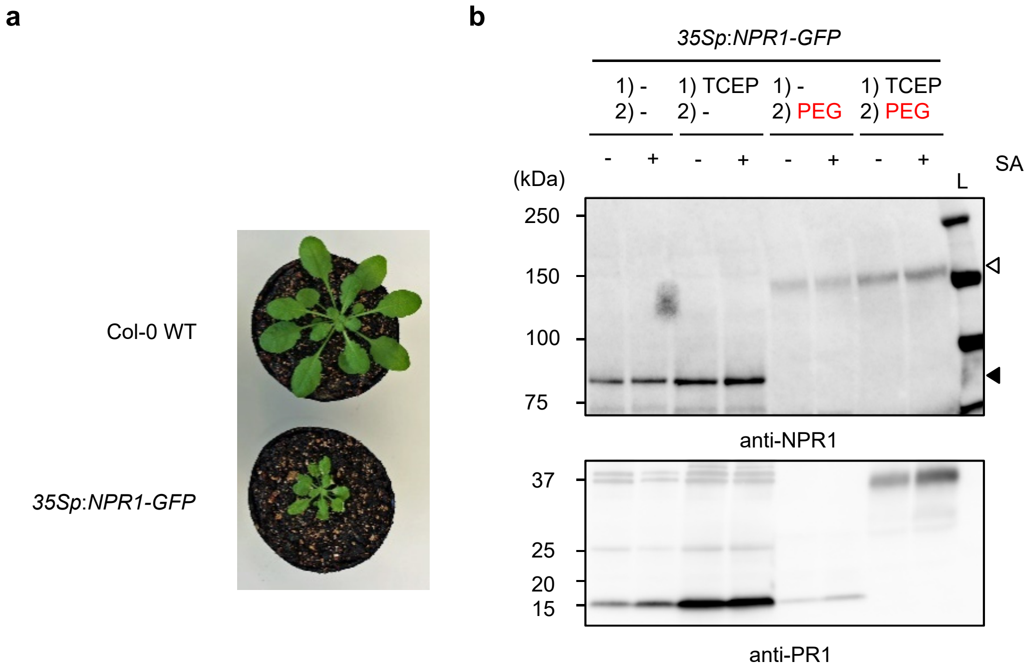

Supplementary Fig. 10 Redox state of the cysteine residues in NPR1-GFP in soil-grown *35Sp*:*NPR1-GFP* plants. a Morphology of 4-week-old Col-0 WT (upper) and *35Sp*:*NPR1-GFP* plants (below). **b** Soil-grown *35Sp*:*NPR1-GFP* plants were treated with 1 mM SA. Proteins were extracted and labeled with 2k PEG-Maleimide (PEG). The labeled proteins were resolved by SDS-PAGE and analyzed by immunoblotting using anti-NPR1 and anti-PR1 antibodies. Black and white arrowheads indicate non-labeled NPR1-GFP (calculated molecular mass: 93 kDa) and PEGylated NPR1-GFP, respectively. Experiments were repeated 3 times with similar results.

Supplementary Table 1 List of NSAIDs identified by chemical screening.

Chemical group	Chemical name	MicroSource	NPDepo
Salicylic acid derivatives	Acetaminosalol	+	
	Acetyl salicylic acid (Aspirine)		+
	D-(-)-Salicin		+
	Salicylic acid		+
Propionic acid derivatives	Fenoprofen	+	
	Flurbiprofen		+
	Ibuprofen	+	+
	Ketoprofen	+	+
	Naproxen*		+
Acetic acid derivatives	Dicrofenac		+
	Indometacin		+
	Sulindac		+
Oxicam derivatives	Isoxicam	+	
	Meloxicam	+	
	Piroxicam		+
	Tenoxicam	+	
Anthranilic acid derivatives	Mefenamic acid		+
Others	Epirizole		+
	Valdecoxib	+	

*It was also present in the MicroSource library but its activity was slightly below the threshold in the first screening.
