# Supplementary material for: Oxicam-type non-steroidal anti-inflammatory drugs inhibit NPR1-mediated salicylic acid pathway
Source: Nat Commun. 2021 Dec 15;12:7303. doi: 10.1038/s41467-021-27489-w (PMC8674334; doi:10.1038/s41467-021-27489-w)
Supplement: Supplementary file 3 — Description of Additional Supplementary Files [file 41467_2021_27489_MOESM3_ESM.pdf]

Description of Additional Supplementary Files

File name: Supplementary Data 1
Description: Normalized expression values of genes in Col-0 WT and npr1-1 treated with SA and/or TNX.

File name: Supplementary Data 2
Description: List of 2,402 genes which were upregulated in Col-0 WT in response to 100 µM TNX treatment for 24 hr (FDR = 0.001 and log2FC > 1).

File name: Supplementary Data 3
Description: List of 2,662 genes which were repressed in Col-0 WT in response to 100 µM TNX treatment for 24 hr (FDR = 0.001 and log2FC < -1).

File name: Supplementary Data 4
Description: List of 940 genes which were upregulated in Col-0 WT in response to 100 µM SA treatment for 24 hr (FDR = 0.001 and log2FC > 1).

File name: Supplementary Data 5
Description: List of 631 genes which were repressed in npr1-1 compared with Col-0 WT in response to 100 µM SA treatment for 24 hr (log2FC (SA/mock in WT) – log2FC (SA/mock in npr1) >1).

File name: Supplementary Data 6
Description: List of 383 genes which were repressed in Col-0 WT co-treated with 100 µM SA + 100 µM TNX compared with Col-0 WT treated with 100 µM SA for 24 hr (log2FC (SA/SA+TNX) > 1).

File name: Supplementary Data 7
Description: Expression levels of the SA-inducible NPR1-dependent genes and group information in Figure 3d.

File name: Supplementary Data 8
Description: List of 207 genes which were repressed in npr1-1 co-treated with 100 µM SA + 100 µM TNX compared with npr1-1 treated with 100 µM SA for 24 hr (log2FC (SA/SA+TNX) in npr1 > 1).

File name: Supplementary Data 9
Description: List of 256 genes which were repressed in Col-0 WT in response to 100 µM SA treatment for 24 hr (FDR = 0.001 and log2FC < -1).

File name: Supplementary Data 10
Description: List of 142 genes which were repressed in Col-0 WT in response to 100 µM SA treatment for 24 hr but were restored in npr1-1 (log2FC (SA/mock in WT)- log2FC (SA/mock in npr1) < -1).

File name: Supplementary Data 11
Description: List of 43 genes which were repressed in Col-0 WT in response to 100 µM SA treatment for 24 hr but were restored by co-treatment with TNX (log2FC (SA/SA+TNX in WT) < -1).
